# Unilateral biportal endoscopic decompression versus anterior cervical decompression and fusion for unilateral cervical radiculopathy or coexisting cervical myelopathy: a prospective, randomized, controlled, noninferiority trial

**DOI:** 10.1186/s12891-024-07697-3

**Published:** 2024-07-25

**Authors:** Wei Peng, Rupeng Chu, Wei Cui, Yin Zhuang, Wenjin Chen, Xiaofei Han, Zhenzhong Sun, Shujun Zhang

**Affiliations:** Department of Spine Surgery, Wuxi Ninth People’s Hospital Affiliated to Soochow University, No. 999 Liangqing Road, Binhu District, Wuxi, Jiangsu China

**Keywords:** Cervical radiculopathy, Cervical myelopathy, Unilateral biportal endoscopy, Anterior cervical decompression and fusion, Discectomy, Spine surgery

## Abstract

**Background:**

Cervical spondylosis (CS), including myelopathy and radiculopathy, is the most common degenerative cervical spine disease. This study aims to evaluate the clinical outcomes of unilateral biportal endoscopy (UBE) compared to those of conventional anterior cervical decompression and fusion (ACDF) for treating unilateral cervical radiculopathy or coexisting cervical myelopathy induced by unilateral cervical herniated discs.

**Methods:**

A prospective, randomized, controlled, noninferiority trial was conducted. The sample consisted of 131 patients who underwent UBE or ACDF was conducted between September 2021 and September 2022. Patients with cervical nerve roots or coexisting spinal cord compression symptoms and imaging-defined unilateral cervical radiculopathy or coexisting cervical myelopathy induced by unilateral cervical herniated discs were randomized into two groups: a UBE group (*n* = 63) and an ACDF group (*n* = 68). The operative time, blood loss, length of hospital stay after surgery, and perioperative complications were recorded. Preoperative and postoperative modified Japanese Orthopaedic Association (mJOA) scale scores, visual analog scale (VAS) scores, neck disability index (NDI) scores, and recovery rate (RR) of the mJOA were utilized to evaluate clinical outcomes.

**Results:**

The hospital stay after surgery was significantly shorter in patients treated with UBE than in those treated with ACDF (*p* < 0.05). There were no significant differences in the neck or arm VAS score, NDI score, mJOA score, or mean RR of the mJOA between the two groups (*p* < 0.05). Only mild complications were observed in both groups, with no significant difference (*p* = 0.30).

**Conclusion:**

UBE can significantly relieve pain and disability without severe complications, and most patients are satisfied with this technique. Consequently, this procedure can be used safely and effectively as an alternative to ACDF for treating unilateral cervical radiculopathy or coexisting cervical myelopathy induced by unilateral cervical herniated discs.

**Trial registration:**

This study was registered in the Chinese Clinical Trial Registry on 02/08/2023 (http://www.chictr.org.cn, #ChiCTR2300074273).

**Supplementary Information:**

The online version contains supplementary material available at 10.1186/s12891-024-07697-3.

## Background

Cervical spondylosis (CS), including myelopathy (CM) and radiculopathy (CR), is the most common degenerative disease of the ageing cervical spine [1]. The spinal cord and nerve roots are often compressed by degenerative and herniated intervertebral discs, cervical spine osteogenic facet joints, and the wrinkled ligamentum flavum (LF), resulting in myelopathy and radiculopathy [2, 3]. Two main symptom complexes are associated with cervical myelopathy and radiculopathy: generalized neck pain or axial neck pain and compression of the cervical spinal cord and nerve roots exiting the cervical spine. It is difficult to determine whether myelopathy or radiculopathy is responsible for the clinical symptoms since myelopathy may mask the symptoms.

Two primary surgical approaches for treating cervical myelopathy and radiculopathy are anterior cervical discectomy and fusion (ACDF) and posterior cervical foraminotomy (PCF). ACDF has been regarded as the standard procedure due to its safety and efficacy for treating cervical myelopathy and radiculopathy. However, ACDF damage to anterior structures is associated with a high risk of postoperative dysphagia and several significant complications, such as adjacent segment diseases, pseudoarthrosis, instrument-related complications, and traditional open posterior cervical laminoplasty or laminectomy damage to the posterior muscles and structures, and carries a high risk of bleeding, nerve injury, neck pain, and progressive cervical kyphosis [4–6].

Minimally invasive cervical surgeries, including percutaneous endoscopic surgery and microscope-assisted surgery, were recently introduced and are the most widely used techniques because they achieve effects similar to those of open surgery and a lower risk of iatrogenic injury [7, 8]. However, the application of these procedures in treating cervical myelopathy and radiculopathy is minimal due to the limited motion of the instruments, small field of view, small space, difficult bleeding control, and steep learning curve [9, 10].

Unilateral biportal endoscopic (UBE) decompression is a novel technique that involves the use of a percutaneous endoscope and has been widely used for treating degenerative diseases of spinal stenosis in recent years [11]. Compared with uniaxial endoscopic approaches, continuous high-definition arthroscope monitoring can perform UBE under a clear and magnified surgical field [12]. Studies have also demonstrated that flexible and unrestricted working tubes improve manoeuvrability and convenience, increasing efficiency and reducing iatrogenic injury [10]. Therefore, it is well accepted that the UBE technique may be superior to uniaxial endoscopy for spinal decompression treatment [13–15].

However, few studies have compared the clinical effectiveness of ACDF with that of UBE. This study aims to provide medical evidence regarding the clinical and radiological outcomes of UBE compared to those of ACDF.

## Materials and methods

### Trial design

We conducted a prospective, randomized, controlled, noninferiority trial at the Wuxi Ninth People’s Hospital Affiliated to Soochow University from September 2021 to September 2022. The patients scheduled to receive UBE surgery or ACDF were randomly assigned in a 1:1 ratio, and the clinical outcomes of the patients who underwent UBE surgery and those who underwent ACDF were compared. The study was approved by the ethics committee of Wuxi Ninth People’s Hospital Affiliated to Soochow University (approval number KS2023019) and was registered in the Chinese Clinical Trial Registry on 02/08/2023 (http://www.chictr.org.cn, #ChiCTR2300074273). We prepared this report in accordance with the Consolidated Standards for Reporting Trials (CONSORT) guidelines.

### Population

We included patients who fulfilled the following inclusion criteria: (1) had symptoms of a unilateral cervical herniated disc at a single level or two adjacent levels that did not improve with conservative treatment, such as numbness, pain, or muscle weakness in the upper extremities with or without gait disturbance for more than 4 weeks; (2) were diagnosed by computed tomography (CT) and magnetic resonance imaging (MRI), which confirmed unilateral cervical herniated discs and the compression of the spinal nerve roots or a combined spinal cord; and (3) had hyperactive reflexes and increased conduction time of somatosensory evoked potentials (SEPs) and motor evoked potentials (MEPs). Patients who met the following exclusion criteria were excluded from the study: (1) without symptoms; (2) complicated with other spinal disorders, such as posterior longitudinal ligament ossification, thoracic spinal stenosis, and lumbar disk herniation; (3) complete paralysis of four limbs; (4) had surgical contraindications or refused surgical treatment; and (5) had cervical spine instability (vertebral body horizontal motion > 3 mm or adjacent intervertebral body angle > 10° based on X-ray flexion-extension motion lateral radiograph). The patient groups were homogeneous in terms of age, sex, BMI, symptom duration, surgical level, and lesion side.

### Randomization and blinding

**Fig. 1:**
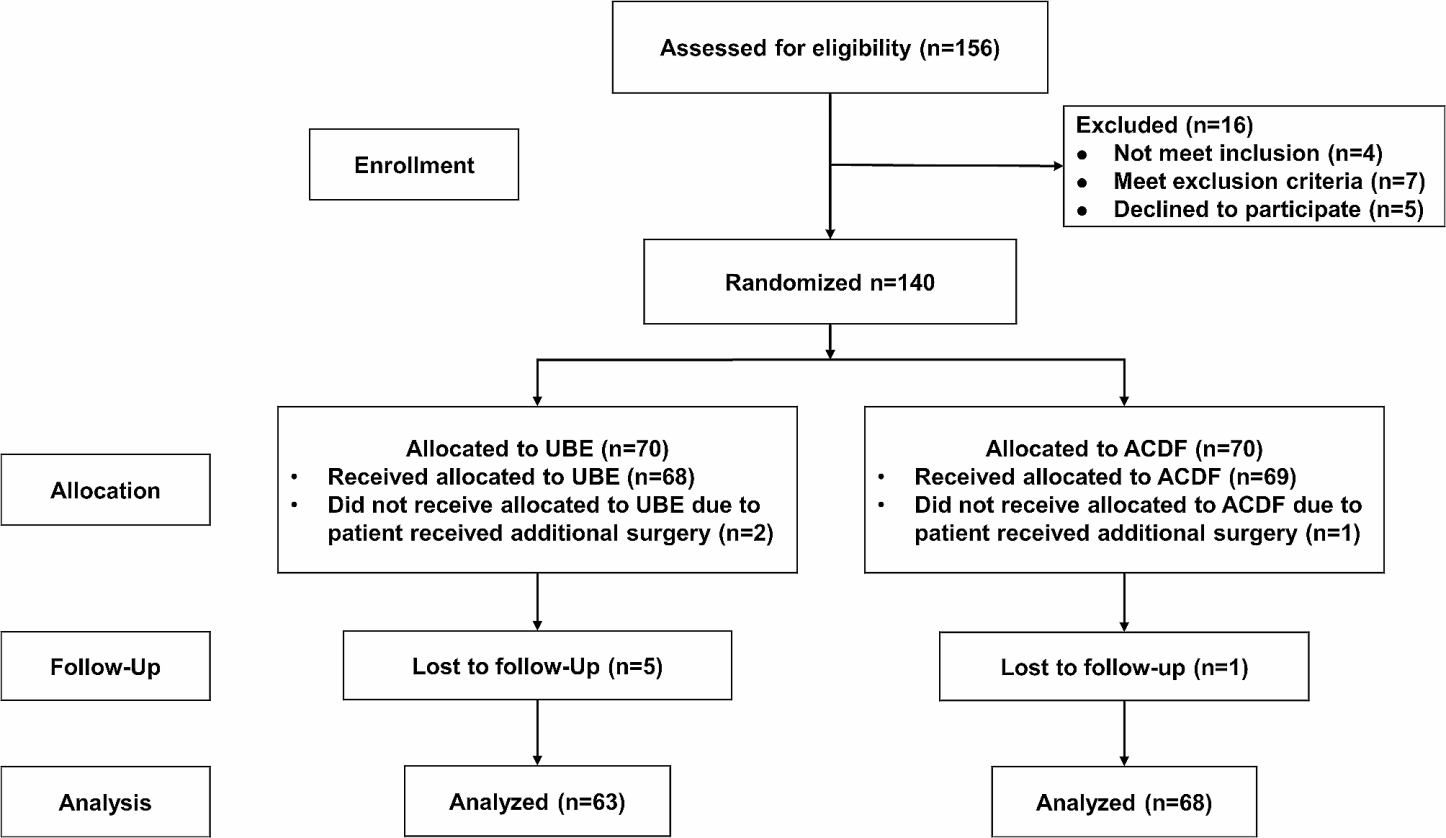
Flow chart of the randomized study

After providing written informed consent, participants were randomly assigned to UBE or ACDF at a 1:1 ratio based on a computer-generated scheme. Random numbers were kept and sealed in envelopes opened on the surgery day. Patient or surgeon blinding was not possible due to the nature of the surgical procedures. However, throughout the trial, the treatment remained concealed for data collectors and statistical analysts, ensuring blinding. All operations were carried out by the same senior surgeon, assisted by four attending physicians who took turns to ensure consistency throughout the study. In addition, none of the four attending physicians participated in collecting preoperative and postoperative data. Details of the subjects included and excluded from the study (from inclusion to analysis) are shown in Fig. 1.

### Intervention

For patients assigned to undergo ACDF, the surgical procedure was primarily based on the Smith–Robinson technique under general anaesthesia. After identifying the appropriate vertebral level, nucleus pulposus forceps were used to dissect the nucleus pulposus. The cartilaginous endplates were removed using a curette. A self-locking stand-alone cage was used as an intervertebral implant following complete dura and nerve root decompression.

**Fig. 2:**
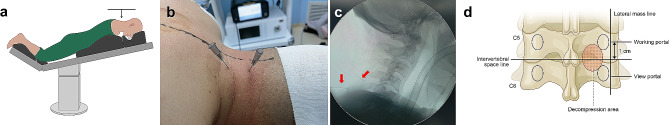
Surgical preparation for the cervical UBE procedure. (**a**) Schematic of the prone position for performing posterior cervical foraminotomy and/or diskectomy with the UBE approach. (**b**) Two 10 ml syringe needles were inserted for the initial approach. (**c**) The exact pathologic location was confirmed with C-arm fluoroscopy in the lateral view. Red arrow, two 10 ml syringe needles. (**d**) Schematic diagram of the portal locations and decompression areas

For patients assigned to undergo UBE, the patient was placed in a prone position on a radiolucent frame for posterior surgery. During this procedure, general anaesthesia was also administered. After the head was secured, the operation table was adjusted so that the targeted intervertebral space was perpendicular to the ground (Fig. 2a). The viewing and working portal sites were placed with 10 ml syringe needles with intraoperative C-arm fluoroscopy in a lateral view for the location of the pathological levels, and the more severe side of compression was selected for the surgical approach (Fig. 2b and c). For procedures involving two pathological segments, the incision was designed to centre at the upper pathological level. It is critical to note that the 10 ml syringe needle was directed towards the vertebrae midway between the two pathological levels. This approach was intended to make it easier to handle the intervertebral disc below. On the anteroposterior view, a vertical line was drawn along the 2 cm paraspinous equivalent to the midline of the lateral mass, while a horizontal line was marked in the intervertebral space. Skin and fascia incisions (1.0 cm long) were made upwards and downwards to create viewing and working portals, respectively (Fig. 2d). Next, the paraspinal muscle was split with a sequential dilator to enlarge the instrument and viewing portal. The precise location of the targeted intervertebral space was determined by fluoroscopy. Semi- sleeve tube was inserted into the surgical incision to create a barrier around the soft tissues, which helps in instrument insertion and allows for a steady water flow.

**Fig. 3:**
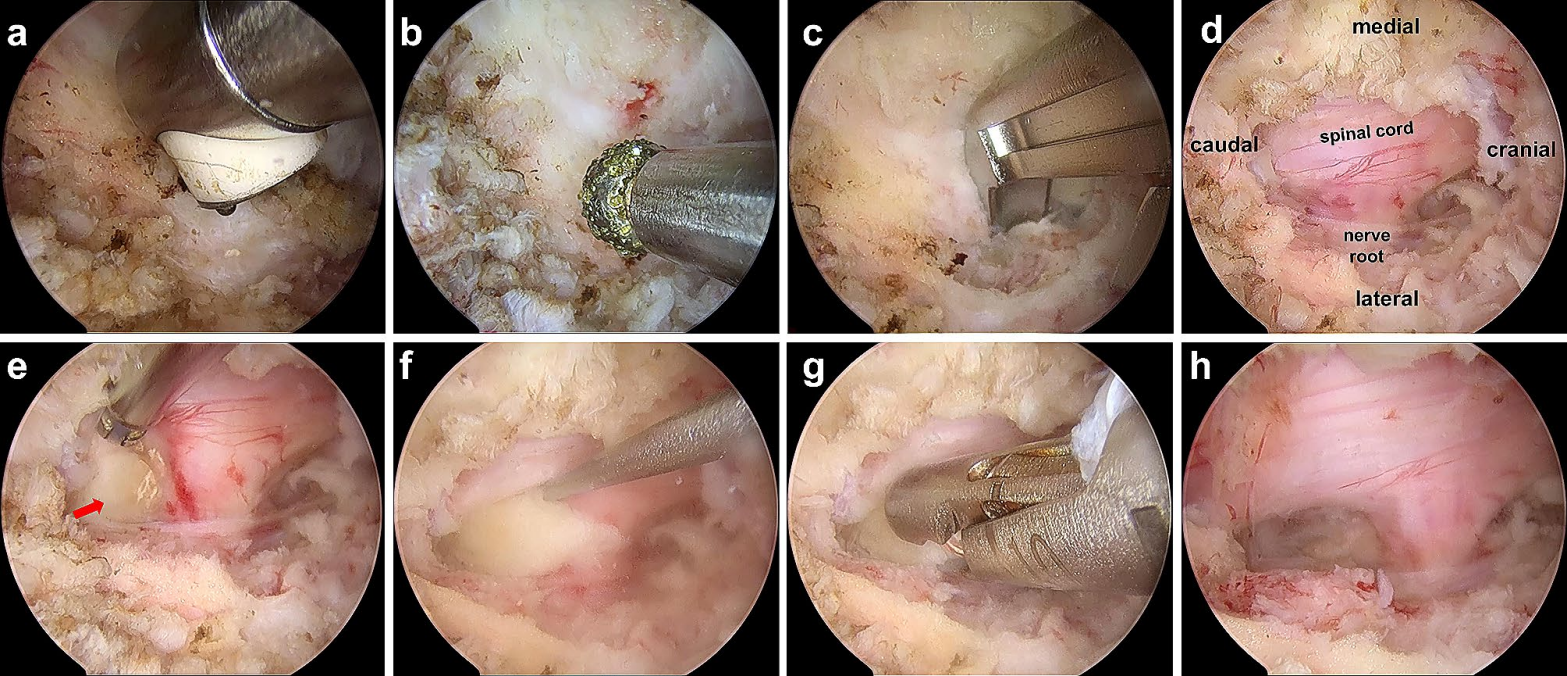
Intraoperative endoscopic images of the UBE procedure. (**a**) Bleeding control and soft tissue detachment was performed with a bipolar radiofrequency instrument. (**b-c**) A high-speed diamond burr and Kerrison rongeurs were used to remove the lamina to expose the attachment of the LF. (**d**) Surgical field after removing the LF. (**e**) The position of the herniated disc (red arrow). (**f-g**) The herniated nucleus pulposus was removed with a small blunt hook and nucleus clamp. (**H**) Full decompression was achieved

Under arthroscopic guidance, bleeding control and soft tissue detachment until the bony surface was reached was performed with a bipolar radiofrequency instrument, and a preliminary workspace was established (Fig. 3a). After exposure of the “V” point, the inferior lamina, superior lamina, laminar interval space, and medial part of the facet joint were dissected sequentially (Fig. 3b). A high-speed diamond burr and Kerrison rongeurs were used to expose the superior and inferior attachments of the LF, which was then removed completely (Fig. 3c and d). Consequently, the decompressing range lies at the junction between the most lateral aspect of the interlaminar space and the most medial part of the facet area. An oval opening of 1.5 to 2 cm was created in the bone. When reaching the cervical cord and the axillary part of the cervical nerve root, the herniated part of the disc was removed with a small blunt hook (Fig. 3e and f). Then, the herniated nucleus pulposus was completely removed (Fig. 3g and h). To ensure complete haemostasis, a hydrostatic test was performed prior to closing the wound. We followed a specific procedure: the saline perfusion was stopped so that when the perfusion water pressure disappeared, we could observe the bleeding point more clearly and perform complete haemostasis at the same time. Before the surgical incision was fully closed, this step was repeated several times until there was no visible bleeding. After complete haemostasis, all instruments were withdrawn, and the wound was closed.

All patients underwent cervical collar fixation for 2 weeks, were discharged from bed 1 day after surgery, and began neck and shoulder functional exercises 2 weeks after surgery.

### Outcome

All patients underwent CT, MRI, X-ray, and electromyography (EMG) scans before surgery. Postoperatively, the patients underwent repeat CT scans to determine the decompression range. The modified Japanese Orthopaedic Association scale (mJOA), visual analog scale (VAS) for upper extremity pain, and neck disability index (NDI) were used to evaluate the efficacy of the treatments preoperatively, postoperatively (3, 6 and 12 months after surgery), and at the final visit [16, 17]. The recovery rate (RR) of the mJOA was calculated by the Hirabayashi method: RR (%) = (postoperative mJOA - preoperative mJOA)/(17 - preoperative mJOA) ×100. According to the RR, surgical results were graded as good (50 ~ 100%), fair (25 ~ 49%), unchanged (0 ~ 24%), or deteriorated (< 0%) [18].

### Sample size and statistical analysis

Based on the arm pain VAS scores as the primary effect measure, the sample size was calculated as follows: Statistical Power = pt(qt(0.025,n-2,0),n-2,-(delta/sigma)/sqrt(4/n)); http://hedwig.mgh.harvard.edu/sample_size/js/js_parallel_quant.html. The treatment response was considered successful when the arm pain intensity decreased by 30% [19]. Using 30% of the mean of one treatment group in terms of a percentile of the other treatment group, a one-tailed significance level of 0.025, and a statistical power of 0.8, the variable calculated was the total number of patients. It is necessary to account for a 10% dropout rate. A parallel design study involving two treatments required 132 participants. The probability was 80% that the investigation would detect a treatment difference at a one-sided 0.025 significance level if the actual difference between the treatments was 0.524 times the standard deviation. As the inclusion rate reached the anticipated level, an interim analysis was not conducted.

All the statistical analyses were conducted using SPSS version 23.0. Descriptive data are presented as the means and SDs. Student’s t tests were used to compare continuous variables between two groups when the data were continuous, normally distributed, and homoscedastic. Chi-square tests and Fisher’s exact tests were applied to evaluate differences between the two groups in other categorical variables. Differences during the postoperative follow-up period in both groups were analysed by one-way variance analysis. *p* < 0.05 was considered to indicate statistical significance.

## Results

### Baseline characteristics

Figure 1 shows the trial enrolment, randomization, and follow-up data. Eventually, 156 patients were enrolled and randomized, with 140 completing the study between September 2021 and September 2022. Of the randomized patients, 131 completed at least 12 months of follow-up and were included in this analysis. Sixty-three patients underwent UBE surgery, and 68 underwent ACDF. Two patients in the UBE group and one in the ACDF group underwent additional surgery and were excluded from the study. Five patients from the UBE group and one patient from the ACDF group were lost during the follow-up period. Table 1 shows the baseline characteristics of the patients. The final follow-up date was September 2023. There were no significant differences in age, sex, BMI, symptom duration, lesion side, or surgical level between the two groups. Surgical-level groups were classified based on the number of discs surgically treated.

**Table 1:** Patient demographics

Characteristic	UBE	ACDF	*p* value
Total patients	63	68	
Age	56.26 ± 12.31	58.31 ± 13.96	0.37
Sex			0.71
Male	41	47	
Female	22	21	
BMI (kg/m^2^)	26.21 ± 3.57	25.75 ± 3.36	0.38
Symptom duration (months)	5.27 ± 1.54	5.17 ± 2.07	0.76
Surgical level			
Single level	35 (55.56%)	42 (61.76%)	0.32
C3/4	7	5	
C4/5	5	13	
C5/6	13	15	
C6/7	10	9	
Two adjacent levels	28 (44.44%)	26 (38.24%)	0.54
C4-6	12	9	
C5-7	11	14	
C6-T1	5	3	
Lesion side			0.60
Left	36	35	
Right	27	33	
Coexisting CM	32	25	0.12

### Perioperative parameters

**Fig. 4:**
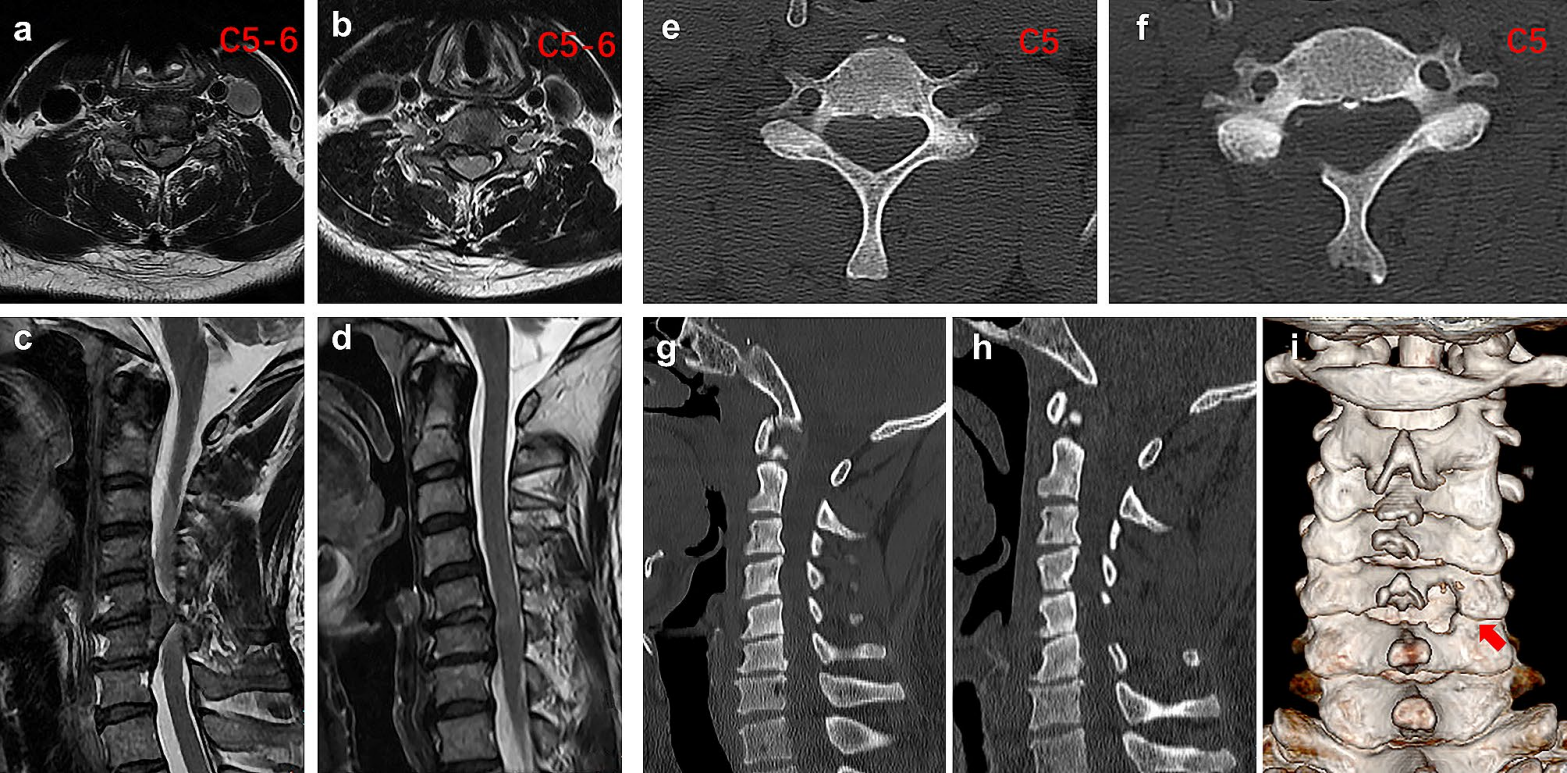
Pre- and postoperative magnetic resonance imaging (MRI) and computed tomography (CT) scans. (**a** and **c**) Preoperative axial and sagittal MRI scans. At the left C5-C6 level, the soft disk protrudes laterally. (**b** and **d**) Postoperative axial and sagittal MRI scans showing that the protruded disk was removed. (**E** and **F**) Preoperative and postoperative axial CT images of the cervical spine. (**G** and **H**) Preoperative and postoperative sagittal CT images of the cervical spine. (**I**) Postoperative 3-dimensional CT image

The mean operation time in the ACDF group was 64.13 ± 28.41 min, which was significantly shorter than that in the UBE group (76.47 ± 32.53 min) (*p* < 0.05). The mean blood loss during the procedure was 62.4 ± 27.8 ml in the ACDF group. The amount of blood loss could not accurately be measured during the UBE procedure. The hospital stay after surgery was 3.50 ± 1.30 days in the UBE group, which was markedly shorter than that in the ACDF group (5.72 ± 1.83 days) (*p* < 0.01) (Table 2). Comparative pre- and postoperative MRI findings in the UBE group confirmed the removal of the disk and release of the compressed area (Fig. 4a-d). Restoration of bone function and decompression in the UBE group was confirmed by computed tomography (CT) (Fig. 4e-i).

**Table 2:** Perioperative parameters

	Operation time (min)	Blood loss	Hospital stay after surgery (days)
UBE	76.47 ± 32.53	-	3.50 ± 1.30
ACDF	64.13 ± 28.41	62.40 ± 27.8	5.72 ± 1.83
t	2.31		7.95
*p*	< 0.05		< 0.01

### Complications

One patient experienced a moderate neurological deficit (muscle strength weakness in the four limbs (3 ~ 4 grades) and unsteady walking), and postoperative MRI revealed that the spinal cord had an increased T2 signal after UBE surgery (Figure S1). Finally, after 10 months of rehabilitation training and hyperbaric oxygen therapy, the patient exhibited a marked improvement with the disappearance of unsteady walking and substantial recovery of limbs’ muscle strength, leaving only weakness (3 ~ 4 grades) in the right upper extremity. Seven patients experienced transient difficulty swallowing after the ACDF procedure. Neck and shoulder pain occurred in six patients in the ACDF group and two patients in the UBE group. These pains disappeared within five days of surgery. No severe complications were detected during the follow-up period except for numbness of the upper extremities in six patients after UBE surgery, which loosened after receiving a small amount of mannitol and glucocorticoids in one to two weeks. Four patients in the ACDF group experienced C5 nerve root palsy, whereas no patients in the UBE group experienced this complication. Postoperative X-rays and MRI of the patients with C5 nerve paralysis showed that they all had satisfactory internal fixation positions, and epidural haematomas were excluded. Methylprednisolone (160 mg) and mannitol (250 ml) were promptly administered intravenously. Patients were advised to engage in active and passive functional exercises at an early stage. No secondary surgery was performed on these patients. At 1 year of follow-up, all patients’ muscle strength returned to grade 5, and their sensory deficits and shoulder pain disappeared. No cerebrospinal fluid cysts or incisional infection occurred during the follow-up. The difference in the complication rates between the two groups was not significant (*p* = 0.12) (Table 3).

**Table 3:** Complications

Complications	ACDF (*n* = 68)	UBE (*n* = 63)	*p* value
Heat injury	0	1	
Transient difficulty swallowing	7	0	
Neck and shoulder pain	6	2	
Upper extremity numbness	0	6	
C5 paralysis	4	0	
**Total**	17	9	0.12

### Clinical outcomes

The preoperative and postoperative VAS scores of the neck and arms, mJOA scores, and NDI scores are shown in Table 4. Immediately after the operation, radicular pain symptoms were significantly reduced for both UBE and ACDF, with similar results in both groups (*p* > 0.05). Based on the mJOA and NDI scores, the cervical function of both groups greatly improved and remained stable during the follow-up period, with no significant differences (*p* > 0.05). In addition, the RR of the mJOA at the last follow-up showed that 37 patients were classified as good and 17 patients were classified as fair in the UBE group; the mean RR was 54.9%. In the ACDF group, 39 patients were classified as good and 19 patients were classified as fair and the mean RR was 57.2%, with no significant difference between the two groups (*p* > 0.05) (Table 5).

**Table 4:** Clinical outcomes

	Pre-surgery	3 months	6 months	12 months	Last follow-up
Neck VAS					
UBE	6.37 ± 0.62	3.22 ± 0.73	2.47 ± 0.48	1.75 ± 0.59	1.72 ± 0.36
ACDF	6.56 ± 0.78	3.13 ± 0.52	2.31 ± 0.64	1.77 ± 0.57	1.62 ± 0.33
t	1.54	0.82	1.61	0.20	1.66
*p*	0.13	0.42	0.11	0.84	0.10
Arm VAS					
UBE	7.35 ± 1.01	2.71 ± 0.63	2.17 ± 0.42	1.52 ± 0.56	1.45 ± 0.53
ACDF	7.33 ± 0.97	2.93 ± 0.82	2.25 ± 0.52	1.58 ± 0.45	1.57 ± 0.67
t	0.12	1.71	0.96	0.68	0.75
*p*	0.91	0.09	0.34	0.50	0.45
mJOA score					
UBE	8.49 ± 1.87	12.07 ± 2.44	13.36 ± 4.26	14.37 ± 3.61	14.81 ± 2.93
ACDF	8.21 ± 2.14	12.66 ± 3.05	14.27 ± 1.87	14.92 ± 4.17	15.16 ± 3.85
t	0.79	1.22	1.60	0.80	0.58
*p*	0.43	0.23	0.11	0.42	0.56
NDI score					
UBE	28.36 ± 9.16	13.13 ± 5.32	10.97 ± 4.52	9.65 ± 3.31	9.36 ± 4.33
ACDF	29.87 ± 10.24	14.37 ± 6.70	9.45 ± 5.54	9.03 ± 3.52	8.87 ± 3.76
t	0.89	1.17	1.71	1.04	0.69
*p*	0.38	0.25	0.09	0.30	0.49

**Table 5:** The recovery rate of mJOA

	Good	Fair	unchanged	deteriorated	RR of mJOA(%)
ACDF	39	19	10	0	57.2
UBE	37	17	8	1	54.9
*p* value					0.75

## Discussion

PCF and ACDF have been indicated to be effective in safely and reliably treating degenerative cervical spine disease. Although these two procedures have diverse applications, they overlap most in terms of their ability to treat unilateral cervical radiculopathy induced by unilateral cervical herniated discs [20]. With the development of minimally invasive surgical techniques in recent years, many of the adverse effects of PCF have been reduced. UBE is a minimally invasive spinal technique that has advanced significantly recently due to its high efficiency, extensive decompression, clear vision, and gentle learning curve [21]. This study compared the clinical effectiveness of ACDF with that of UBE in treating unilateral cervical radiculopathy or cervical myelopathy induced by unilateral cervical herniated discs.

Decompression of spinal stenosis is one of the core features of UBE [10, 22, 23]. Recent studies have shown that compared with uniportal endoscopy and microsurgery, UBE involves thorough decompression and more articular process retention, and its range and degree are similar to those of open surgery [15]. Moreover, UBE can relieve compression from the lamina’s ventral and contralateral sides via an over-the-top approach, which cannot be performed in open surgery [23]. In the present study, compared with preoperative CT and MRI, postoperative imaging showed that the compressors, such as the herniated disc, osteogenic facet joints, and wrinkled LF, were removed, an adequate decompression area was achieved, and no significant spinal instability was observed.

In most patients, UBE surgery improves neurological function and reduces pain. Our data showed that the neck and arm VAS score, mean mJOA score, NDI score and average RR of the mJOA in the UBE group were not significantly different from those in the ACDF group after the operation. A longer follow-up with more patients is necessary to confirm the outcomes. Nevertheless, it is well known that neurological recovery will be further enhanced, and a greater RR is likely to occur with a longer follow-up period. Additionally, minimally invasive PCF may be associated with lower medical expenses than ACDF. The above results show that UBE is an effective alternative for treating cervical lateral soft disks and spinal stenosis.

High efficiency and flexibility are other characteristics of UBE. Unlike uniaxial endoscopic approaches, UBE has viewing and working portals that can be manoeuvred independently. The three-dimensional view and magnified surgical field provided by arthroscopy, similar to posterior cervical diskectomy, enables surgeons to carry out this procedure more meticulously and prevent unnecessary injuries [24]. Moreover, the free working space and universal applicability of these instruments, including drills, Kerrison punches, and pituitary devices, which are applied in traditional open surgery, enable surgeons to achieve greater efficiency in haemostasis and decompression of spinal stenosis [23]. However, UBE is time-consuming to perform. In this study, the operative time of the UBE group was longer than that of the ACDF group.

Furthermore, prolonged time in the prone position and the use of an invasive fixation device may increase the risk of associated morbidity during traditional PCF. Blood loss during the UBE procedure cannot be accurately measured, but it is intuitively lower than that of ACDF. These results suggest that UBE can achieve favourable outcomes with less blood loss and a shorter postoperative length of stay.

Despite the excellent results of ACDF, some significant complications, such as adjacent segment diseases, pseudoarthrosis, and instrument-related complications, which may require reoperation, remain challenging [25]. PCF preserves motion, and graft-related complications are avoided. Thus, this procedure has the advantage of a lower incidence of same- and adjacent-segment disease than ACDF [26]. However, a principal drawback of traditional open PCF is the extensive damage caused to the paraspinal muscles and facet joint injury, leading to intersegmental instability, neck pain, muscle spasms, and progressive cervical kyphosis after surgery. In our study, most patients experienced neck pain relief, and no patients experienced aggravated neck pain, muscle spasms, or intersegmental instability during follow-up after UBE surgery. This may be because the UBE procedure minimizes bone removal and muscle disruption without compromising decompression, resulting in little loss of spine stability [14]. C5 nerve root palsy is another common complication of posterior cervical open surgery, especially in double-door cervical laminoplasty [27–29]. No patient experienced C5 nerve root palsy after UBE in this study. However, C5 nerve paralysis was present on the second day after ACDF surgery in four patients. These patients typically had unilateral symptomatic manifestations of biceps brachii muscle paralysis (one patient whose muscle strength was grade 3) and deltoid muscle paralysis (three patients whose muscle strength ranged from grade 3 ~ 4), and all four experienced mild numbness in the area innervated by the C5 nerve root as well as shoulder pain. This may be because the cervical cord migrates and rotates asymmetrically within the spinal canal because of asymmetric decompression on the left or right side during ACDF surgery [30]. A unilateral increase in C5 nerve root tone may result in C5 nerve paralysis. These results suggest that UBE surgery may be more suitable than ACDF as a first-line treatment.

However, thermal damage to the spinal cord and nerve root caused by the use of a bipolar radiofrequency probe has not been reported to be a complication of the UBE procedure. In this study, one patient experienced spinal cord thermal damage and had a moderate neurological deficit after UBE surgery. We did not touch this patient’s spinal cord or nerve root during surgery, but postoperative MRI showed that the spinal cord had an increased T2 signal. Thermal damage to the spinal cord and nerve roots may be associated with the tiny incision obstructing saline output and overusing the bipolar radiofrequency probe, resulting in the heat generated by bipolar coagulation not being removed in time. The patient’s upper limb weakness recovered moderately, and he recovered after 10 months of rehabilitation training and hyperbaric oxygen therapy. Our experience shows that a semi-sleeve tube, a unique instrument for UBE surgery that is inserted into the working portal to assist in saline discharge, can significantly reduce the risk of thermal damage to the spinal cord and nerve roots.

In summary, based on our surgical experience, we concluded that the potential indications for cervical UBE surgery are (1) hypertrophy or folding of the posterior LF, or compression-causing material primarily located on the posterior side of the spinal canal, compressing the spinal cord in one or two segments; (2) narrowing of the nerve root canal causing compression or irritation of the nerve root; (3) unilateral herniated discs of one or two segments, compressing the spinal cord and the nerve root; and (4) young patients with a single- or two-segment central disc herniation that is not severe, for whom conservative treatment is ineffective and who want surgical treatment but refuse fusion surgery. It is critical to note that the presence of more than three segments that require processing and large, calcified, or ossified compression-causing materials that originate on the anterior side are relative contraindications to cervical UBE surgery.

### Limitations

This research has several limitations that need to be considered. First, in this study, complications such as axial symptoms and C5 palsy were uncommon, so comparisons of complications between groups may have been biased due to the low incidence of complications. Moreover, we were unable to assess the efficacy of our treatment after a two-year follow-up period, which might not be long enough to evaluate long-term effects. In addition, this was a single-centre prospective study, which might not represent the universal population.

## Conclusion

In summary, our preliminary study suggested that the UBE decompression technique for cervical decompression is highly effective, safe, and effective for unilateral cervical radiculopathy or coexisting cervical myelopathy induced by unilateral cervical herniated discs.

### Electronic supplementary material

Below is the link to the electronic supplementary material.

Supplementary Material 1

Supplementary Material 2

Supplementary Material 3

## Data Availability

The dataset generated during this study is available from the corresponding author (SJZ) upon reasonable request.
