# Supplementary material for: Unilateral biportal endoscopic decompression versus anterior cervical decompression and fusion for unilateral cervical radiculopathy or coexisting cervical myelopathy: a prospective, randomized, controlled, noninferiority trial
Source: BMC Musculoskelet Disord. 2024 Jul 25;25:582. doi: 10.1186/s12891-024-07697-3 (PMC11270769; doi:10.1186/s12891-024-07697-3)
Supplement: Supplementary file 1 — Supplementary Material 1 [file 12891_2024_7697_MOESM1_ESM.docx]

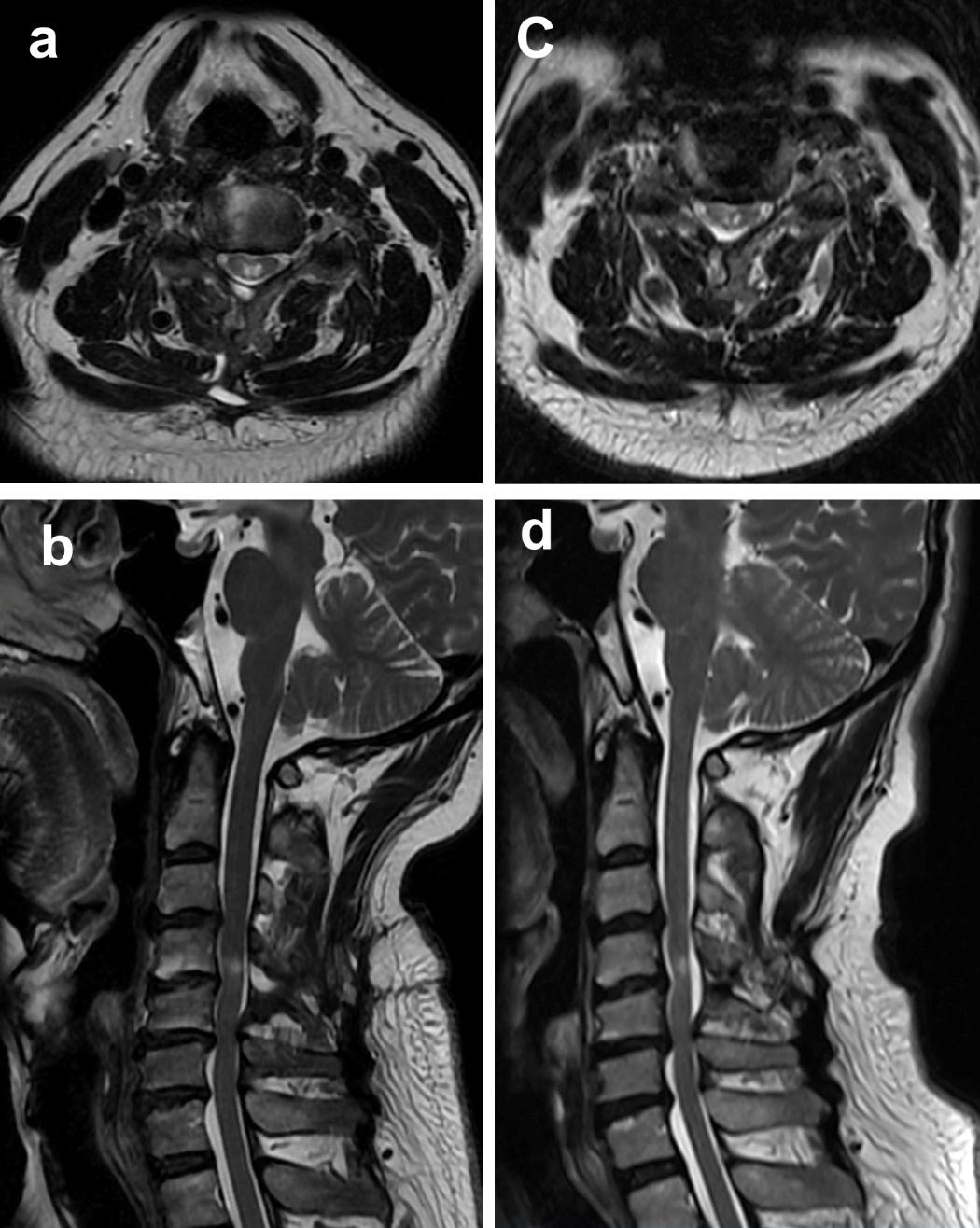


**Figure S1** MRI of the patient with heat injury at two months post-surgery and 1-year follow-up. (a and b) Axial and sagittal MRI scans at two months post-surgery. At the C4-5 level, the spinal cord had an increased T2 signal. (c and d) 1-year follow-up axial and sagittal MRI scans.
