# Supplementary material for: Unilateral biportal endoscopic decompression versus anterior cervical decompression and fusion for unilateral cervical radiculopathy or coexisting cervical myelopathy: a prospective, randomized, controlled, noninferiority trial
Source: BMC Musculoskelet Disord. 2024 Jul 25;25:582. doi: 10.1186/s12891-024-07697-3 (PMC11270769; doi:10.1186/s12891-024-07697-3)
Supplement: Supplementary file 3 — Supplementary Material 3 [file 12891_2024_7697_MOESM3_ESM.docx]

**Research Protocol**

**Project summary**

**Objectives:** Cervical spondylosis (CS), including myelopathy and radiculopathy, is the most common degenerative cervical spine disease. This study aims to evaluate the clinical outcomes of unilateral biportal endoscopy (UBE) compared to those of conventional anterior cervical decompression and fusion (ACDF) for treating unilateral cervical radiculopathy or coexisting cervical myelopathy induced by unilateral cervical herniated discs.

**Methods**

A prospective, randomized, controlled, noninferiority trial was conducted. The sample consisted of 131 patients who underwent UBE or ACDF was conducted between September 2021 and September 2022. Patients with spinal cord compression symptoms and imaging-defined unilateral cervical radiculopathy or coexisting cervical myelopathy induced by unilateral cervical herniated discs were randomized into two groups: a UBE group (n=63) and an ACDF group (n=68). The operative time, blood loss, length of hospital stay after surgery, and perioperative complications were recorded. Preoperative and postoperative modified Japanese Orthopaedic Association (mJOA) scale scores, visual analog scale (VAS) scores, neck disability index (NDI) scores, and recovery rate (RR) of the mJOA were utilized to evaluate clinical outcomes.

**Population**

Between September 2021 and September 2022, patients with spinal cord compression symptoms and imaging-defined unilateral cervical radiculopathy were enrolled in this study and treated with UBE decompression and ACDF.

**Time frame:**

All participants underwent UBE decompression or ACDF surgery from September 2021 to September 2022. And then, we followed up with them 3, 6, and 12 months after surgery. We recorded the modified Japanese Orthopaedic Association scale (mJOA) and Visual Analog Scale (VAS) for upper extremities pain and Neck Disability Index (NDI) to evaluate the efficacy of UBE and ACDF surgery. The final follow-up date was September 2023.

**Expected outcomes:**

UBE can significantly relieve pain and disability without severe complications, and most patients are satisfied with this technique. Consequently, this procedure can be used safely and effectively as an alternative to ACDF for treating unilateral cervical radiculopathy or coexisting cervical myelopathy induced by unilateral cervical herniated discs.

**General information**

**Protocol title：**

Unilateral Biportal Endoscopic Decompression versus Anterior Cervical Decompression and Fusion for Unilateral Cervical Radiculopathy or Coexisting Cervical Myelopathy: A Prospective, Randomized, Controlled, Noninferiority Trial

**Funder name:**

This work was supported by grants from the General Project of Wuxi Municipal Health Commission in 2020 (grant number M202002) and the Science and Technology Bureau of Wuxi (Nos. K20221063 and Y20222029).

**Clinical institute:** The study was conducted at Wuxi Ninth People's Hospital Affiliated to Soochow University.

**Trial registration:** This study was registered in the Chinese Clinical Trial Registry on 02/08/2023 (http://www.chictr.org.cn, #ChiCTR2300074273).

**Rationale & background information**

Cervical spondylosis (CS), including myelopathy and radiculopathy, is the most common degenerative disease of the ageing cervical spine[1]. The spinal cord and nerve roots are often compressed by degenerative and herniated intervertebral discs, cervical spine osteogenic facet joints, and the wrinkled ligamentum flavum (LF), resulting in myelopathy and radiculopathy [2, 3]. Two main symptom complexes are associated with cervical myelopathy and radiculopathy: generalized neck pain or axial neck pain and compression of the cervical spinal cord and nerve roots exiting the cervical spine. It is difficult to determine whether myelopathy or radiculopathy is responsible for the clinical symptoms since myelopathy may mask the symptoms.

Two primary surgical approaches for treating cervical myelopathy and radiculopathy are anterior cervical discectomy and fusion (ACDF) and posterior cervical foraminotomy (PCF). ACDF has been regarded as the standard procedure due to its safety and efficacy for treating cervical myelopathy and radiculopathy. However, ACDF damage to anterior structures is associated with a high risk of postoperative dysphagia and several significant complications, such as adjacent segment diseases, pseudoarthrosis, instrument-related complications, and traditional open posterior cervical laminoplasty or laminectomy damage to the posterior muscles and structures, and carries a high risk of bleeding, nerve injury, neck pain, and progressive cervical kyphosis[4-6].

Minimally invasive cervical surgeries, including percutaneous endoscopic surgery and microscope-assisted surgery, were recently introduced and are the most widely used techniques because they achieve effects similar to those of open surgery and a lower risk of iatrogenic injury[7, 8]. However, the application of these procedures in treating cervical myelopathy and radiculopathy is minimal due to the limited motion of the instruments, small field of view, small space, difficult bleeding control, and steep learning curve[9, 10].

Unilateral biportal endoscopic (UBE) decompression is a novel technique that involves the use of a percutaneous endoscope and has been widely used for treating degenerative diseases of spinal stenosis in recent years[11]. Compared with uniaxial endoscopic approaches, continuous high-definition arthroscope monitoring can perform UBE under a clear and magnified surgical field[12]. Studies have also demonstrated that flexible and unrestricted working tubes improve manoeuvrability and convenience, increasing efficiency and reducing iatrogenic injury[10]. Therefore, it is well accepted that the UBE technique may be superior to uniaxial endoscopy for spinal decompression treatment[13-15].

**Study goals and objectives**

There were few studies compared the clinical effectiveness of ACDF with UBE. The purpose of this study is to provide evidence-based medical evidence regarding the clinical and radiological outcomes of UBE in comparison to ACDF.

**Study design**

**Trail design**

We conducted a prospective, randomized, controlled, noninferiority trial at the Wuxi Ninth People’s Hospital Affiliated to Soochow University from September 2021 to September 2022. The patients scheduled to receive UBE surgery or ACDF were randomly assigned in a 1:1 ratio, and the clinical outcomes of the patients who underwent UBE surgery and those who underwent ACDF were compared. The study was approved by the ethics committee of Wuxi Ninth People’s Hospital Affiliated to Soochow University (approval number KS2023019) and was registered in the Chinese Clinical Trial Registry on 02/08/2023 (http://www.chictr.org.cn, #ChiCTR2300074273). We prepared this report in accordance with the Consolidated Standards for Reporting Trials (CONSORT) guidelines.

**Population**

We included patients who fulfilled the following inclusion criteria: (1) had systemic symptoms of a unilateral cervical herniated disc at a single level or two adjacent levels that did not improve with conservative treatment, such as numbness, pain, or muscle weakness in the upper extremities for more than 4 weeks without gait disturbance; (2) were diagnosed by computed tomography (CT) and magnetic resonance imaging (MRI), which confirmed unilateral cervical herniated discs and the compression of the spinal nerve roots or a combined spinal cord; and (3) had hyperactive reflexes and increased conduction time of somatosensory evoked potentials (SEPs) and motor evoked potentials (MEPs). Patients who met the following exclusion criteria were excluded from the study: (1) without symptoms; (2) complicated with other spinal disorders, such as posterior longitudinal ligament ossification, thoracic spinal stenosis, and lumbar disk herniation; (3) complete paralysis of four limbs; (4) had surgical contraindications or refused surgical treatment; and (5) had cervical spine instability (vertebral body horizontal motion >3 mm or adjacent intervertebral body angle >10° based on X-ray flexion-extension motion lateral radiograph). The patient groups were homogeneous in terms of age, sex, BMI, symptom duration, surgical level, and lesion side.

**Randomization and blinding**

After providing written informed consent, participants were randomly assigned to UBE or ACDF at a 1:1 ratio based on a computer-generated scheme. Random numbers were kept and sealed in envelopes opened on the surgery day. Patient or surgeon blinding was not possible due to the nature of the surgical procedures. However, throughout the trial, the treatment remained concealed fro data collectors and statistical analysts, ensuring blinding. All operations were carried out by the same senior surgeon, assisted by four attending physicians who took turns to ensure consistency throughout the study. In addition, none of the four attending physicians participated in collecting preoperative and postoperative data. Details of the subjects included and excluded from the study (from inclusion to analysis) are shown in Fig. 1.

**Methodology**

**Intervention**

For patients assigned to undergo ACDF, the surgical procedure was primarily based on the Smith–Robinson technique under general anaesthesia. After identifying the appropriate vertebral level, nucleus pulposus forceps were used to dissect the nucleus pulposus. The cartilaginous endplates were removed using a curette. A self-locking stand-alone cage was used as an intervertebral implant following complete dura and nerve root decompression.

For patients assigned to undergo UBE, the patient was placed in a prone position on a radiolucent frame for posterior surgery. During this procedure, general anaesthesia was also administered. After the head was secured, the operation table was adjusted so that the targeted intervertebral space was perpendicular to the groun. The viewing and working portal sites were placed with 10 ml syringe needles with intraoperative C-arm fluoroscopy in a lateral view for the location of the pathological levels, and the more severe side of compression was selected for the surgical approac. For procedures involving two pathological segments, the incision was designed to centre at the upper pathological level. It is critical to note that the 10 ml syringe needle was directed towards the vertebrae midway between the two pathological levels. This approach was intended to make it easier to handle the intervertebral disc below. On the anteroposterior view, a vertical line was drawn along the 2 cm paraspinous equivalent to the midline of the lateral mass, while a horizontal line was marked in the intervertebral space. Skin and fascia incisions (1.0 cm long) were made upwards and downwards to create viewing and working portals, respectively. Next, the paraspinal muscle was split with a sequential dilator to enlarge the instrument and viewing portal. The precise location of the targeted intervertebral space was determined by fluoroscopy.

Under arthroscopic guidance, bleeding control and soft tissue detachment until the bony surface was reached was performed with a bipolar radiofrequency instrument, and a preliminary workspace was established. After exposure of the “V” point, the inferior lamina, superior lamina, laminar interval space, and medial part of the facet joint were dissected sequentiall. A high-speed diamond burr and Kerrison rongeurs were used to expose the superior and inferior attachments of the LF, which was then removed completely. Consequently, the decompressing range lies at the junction between the most lateral aspect of the interlaminar space and the most medial part of the facet area. An oval opening of 1.5 to 2 cm was created in the bone. When reaching the cervical cord and the axillary part of the cervical nerve root, the herniated part of the disc was removed with a small blunt hook. Then, the herniated nucleus pulposus was completely remove. To ensure complete haemostasis, a hydrostatic test was performed prior to closing the wound. We followed a specific procedure: the saline perfusion was stopped so that when the perfusion water pressure disappeared, we could observe the bleeding point more clearly and perform complete haemostasis at the same time. Before the surgical incision was fully closed, this step was repeated several times until there was no visible bleeding. After complete haemostasis, all instruments were withdrawn, and the wound was closed.

All patients underwent cervical collar fixation for 2 weeks, were discharged from bed 1 day after surgery, and began neck and shoulder functional exercises 2 weeks after surgery.

**Outcome**

All patients underwent CT, MRI, X-ray, and electromyography (EMG) scans before surgery. Postoperatively, the patients underwent repeat CT scans to determine the decompression range. The modified Japanese Orthopaedic Association scale (mJOA), visual analog scale (VAS) for upper extremity pain, and neck disability index (NDI) were used to evaluate the efficacy of the treatments preoperatively, postoperatively (3, 6 and 12 months after surgery), and at the final visit[16, 17]. The recovery rate (RR) of the mJOA was calculated by the Hirabayashi method: RR (%) = (postoperative mJOA - preoperative mJOA)/(17 - preoperative mJOA) ×100. According to the RR, surgical results were graded as good (50~100%), fair (25~49%), unchanged (0~24%), or deteriorated (< 0%)[18].

**Safety considerations**

All operations were carried out by the same senior surgeon, assisted by four attending physicians who took turns to ensure consistency throughout the study. In addition, none of the four attending physicians participated in collecting preoperative and postoperative data. We have established a comprehensive standard operating procedure for adverse and severe adverse events. Specifically, as follows:

1. **Treatment of adverse events**

Treatment: Depending on the nature and extent of the adverse events that occur, the investigator should treat them promptly and make a preliminary determination of causality, relevance, and degree of damage to the RCTs, including a decision on the need to discontinue the clinical trial.

**Record:** The investigator should fill in the Adverse Event Record Form, recording the description of the adverse event and all related symptoms, time of occurrence, time of termination, degree and frequency of episodes, tests performed as a result of the adverse event, whether treatment is required (if so, record the treatment given), the outcome of the adverse event, the relationship with the RCTs, and whether measures are taken concerning the RCTs (e.g., discontinuing their use, removing them, etc.), to ensure the accuracy, completeness and accuracy of the record. Ensure the form is true, accurate, complete, and timely, signed and dated.

**Follow-up:** All adverse events should be tracked and investigated until they are adequately resolved, or the condition stabilizes. Various tracking and follow-up methods can be chosen depending on the severity of the adverse event, including hospitalizations, outpatient visits, home visits, telephone calls, etc.

1. **Treatment of serious adverse events**

Treatment: The investigator, the principal investigator, or the receiving physician of the emergency shall be the first responsible person to decide whether treatment is needed according to the specific circumstances of the occurrence. If the situation is urgent, the necessary treatment measures shall be taken immediately. If serious adverse events cannot be handled by the department independently, the relevant department will be contacted in time for consultation and joint treatment; in an emergency, the medical staff will be sent to the department's ICU.

**Follow-up**

We followed up with them 3, 6, and 12 months after surgery. We recorded the modified Japanese Orthopaedic Association scale (mJOA) and Visual Analog Scale (VAS) for upper extremities pain and Neck Disability Index (NDI) to evaluate the efficacy of UBE and ACDF surgery. The final follow-up date was September 2023.

During the follow-up period, if the patient develops a complication, the name of the complication, the time of occurrence, the examination results, the measures taken, and the follow-up time need to be recorded in detail.

**Data management and statistical analysis**

**Sample size and statistical analysis**

Based on the arm pain VAS scores as the primary effect measure, the sample size was calculated as follows: Statistical Power=pt(qt(.025,n-2,0),n-2,-(delta/sigma)/sqrt(4/n)); http://hedwig.mgh.harvard.edu/sample_size/js/js_parallel_quant.html. The treatment response was considered successful when the arm pain intensity decreased by 30%[19]. Using 30% of the mean of one treatment group in terms of a percentile of the other treatment group, a one-tailed significance level of 0.025, and a statistical power of 0.8, the variable calculated was the total number of patients. It is necessary to account for a 10% dropout rate. A parallel design study involving two treatments required 132 participants. The probability was 80% that the investigation would detect a treatment difference at a one-sided 0.025 significance level if the actual difference between the treatments was 0.524 times the standard deviation. As the inclusion rate reached the anticipated level, an interim analysis was not conducted.

All the statistical analyses were conducted using SPSS version 23.0. Descriptive data are presented as the means and SDs. Student's t tests were used to compare continuous variables between two groups when the data were continuous, normally distributed, and homoscedastic. Chi-square tests and Fisher's exact tests were applied to evaluate differences between the two groups in other categorical variables. Differences during the postoperative follow-up period in both groups were analysed by one-way variance analysis. *p*< 0.05 was considered to indicate statistical significance.

**Quality assurance**

A professional quality controller with specific clinical trial experience and familiar with the requirements of RCTs not related to the undertaking of this research project is assigned by the person in charge of this specialty to be responsible for the implementation of quality control and supervision of all aspects of this research, including pre-trial, mid-trial and post-trial. The principal investigator shall supervise and control the experiment. When the trial project is initiated, the professional group of the trial shall organize and hold an initiation meeting to train all the participants in the clinical trial and record the training. The quality controller shall participate in the initiation meeting to learn about the trial program and process. He shall also inform researchers of trial implementation requirements and precautions. Professional quality controllers should review trial data promptly to ensure accurate and reliable results.

The quality controller shall strictly implement GCP and comply with relevant national laws and regulations. He shall carry out quality control according to the trial program and ensure sufficient time to control the whole clinical trial process. Understand the clinical trial's progress and identify any problems found during the test. To ensure timely improvements, report them promptly to the principal investigator, the professional responsible, and the institution's office. Strictly review and examine the records of enrollment criteria, test examinations, follow-up data, etc., of each case per the trial protocol requirements, and promptly communicate with the investigators about any problems. Check the outpatient or inpatient medical records of the subjects to confirm that the source documents recorded by the investigator are valid, accurate, and complete; check the consistency between the source documents and the data and ensure that the data obtained are derived from the source documents and are consistent with the source documents.

**Expected outcomes of the study**

Unilateral biportal endoscopic (UBE) decompression is a novel technique operated through a percutaneous endoscope, which has been widely used for degenerative diseases of spinal stenosis in recent years. Current applications in cervical spine disorders are mainly applied to unilateral CR. However, there were few studies focused on the clinical effectiveness unilateral CR, especially studies of RCTs compared to ACDF. The purpose of this study is to provide evidence-based medical evidence regarding the clinical and radiological outcomes of UBE in comparison to ACDF.

**Duration of the project**

This project study lasts 2 years from September 2021 to September 2023. The All participants underwent UBE decompression or ACDF surgery from September 2021 to September 2022. And then, we followed up with them 3, 6, and 12 months after surgery. The final follow-up date was September 2023.

**Project management**

SJZ participated in the study conception and design, performed the UBE and ACDF surgeries for the participants, and revised the drafted manuscript. WP participated in the study conception and design, applied the taping protocol of the study, enrolled patients, performed the statistical analyses, and drafted the manuscript. RPC generated the random allocation sequence and participated in the data acquisition and data analyses. WC participated in the study design, allocation of the participants to the interventions, and data acquisition. YZ participated in the study design and data acquisition. WJC participated in the study design and statistical analyses. XFH participated in the study design and data acquisition. ZZS participated in the study design and revised the draft manuscript. All the authors have read and approved the final manuscript.

**Ethics**

Ethical approval was obtained from the ethics committee of Wuxi Ninth People’s Hospital Affiliated to Soochow University (approval number KS2023019). All procedures were performed in accordance with the ethical standards of the Ethics Committee of the hospital, and under the 1964 Helsinki Declaration and its later amendments or comparable ethical standards. This project was registered in the Chinese Clinical Trial Registry on 02/08/2023 (http://www.chictr.org.cn,#ChiCTR2300074273). All patients signed an informed consent form prior to their participation in the study.

**Research protocol: part 2**

**Other support for the project**

None.

**Collaboration with other scientists or research institutions**

None.

**Links to other projects**

None.

**Other research activities of the investigators**

None.

**Financing and insurance**

None.

1. Theodore N: **Degenerative Cervical Spondylosis**. *N Engl J Med* 2020, **383**(2):159-168.

2. Cook C, Roman M, Stewart KM, Leithe LG, Isaacs R: **Reliability and diagnostic accuracy of clinical special tests for myelopathy in patients seen for cervical dysfunction**. *J Orthop Sports Phys Ther* 2009, **39**(3):172-178.

3. Sakaura H, Hosono N, Mukai Y, Ishii T, Yoshikawa H: **C5 palsy after decompression surgery for cervical myelopathy: review of the literature**. *Spine (Phila Pa 1976)* 2003, **28**(21):2447-2451.

4. Asher AL, Devin CJ, Kerezoudis P, Chotai S, Nian H, Harrell FE, Sivaganesan A, McGirt MJ, Archer KR, Foley KT *et al*: **Comparison of Outcomes Following Anterior vs Posterior Fusion Surgery for Patients With Degenerative Cervical Myelopathy: An Analysis From Quality Outcomes Database**. *Neurosurgery* 2019, **84**(4):919-926.

5. Puvanesarajah V, Jain A, Shimer AL, Singla A, Shen F, Hassanzadeh H: **Complications and Mortality Following One to Two-Level Anterior Cervical Fusion for Cervical Spondylosis in Patients Above 80 Years of Age**. *Spine* 2017, **42**(9):E509-E514.

6. Sakai K, Yoshii T, Hirai T, Arai Y, Torigoe I, Tomori M, Sato H, Okawa A: **Cervical Sagittal Imbalance is a Predictor of Kyphotic Deformity After Laminoplasty in Cervical Spondylotic Myelopathy Patients Without Preoperative Kyphotic Alignment**. *Spine* 2016, **41**(4):299-305.

7. Gala VC, O'Toole JE, Voyadzis JM, Fessler RG: **Posterior minimally invasive approaches for the cervical spine**. *Orthop Clin North Am* 2007, **38**(3):339-349; abstract v.

8. Winder MJ, Thomas KC: **Minimally invasive versus open approach for cervical laminoforaminotomy**. *Can J Neurol Sci* 2011, **38**(2):262-267.

9. Oshima Y, Takeshita K, Inanami H, Takano Y, Koga H, Iwahori T, Baba S, Tanaka S: **Cervical microendoscopic interlaminar decompression through a midline approach in patients with cervical myelopathy: a technical note**. *J Neurol Surg A Cent Eur Neurosurg* 2014, **75**(6):474-478.

10. Merter A, Karaeminogullari O, Shibayama M: **Comparison of Radiation Exposure Among 3 Different Endoscopic Diskectomy Techniques for Lumbar Disk Herniation**. *World Neurosurg* 2020, **139**:e572-e579.

11. Hwa Eum J, Hwa Heo D, Son SK, Park CK: **Percutaneous biportal endoscopic decompression for lumbar spinal stenosis: a technical note and preliminary clinical results**. *Journal of Neurosurgery Spine* 2016, **24**(4):602-607.

12. Park JH, Jun SG, Jung JT, Lee SJ: **Posterior Percutaneous Endoscopic Cervical Foraminotomy and Diskectomy With Unilateral Biportal Endoscopy**. *Orthopedics* 2017, **40**(5):e779-e783.

13. Aygun H, Abdulshafi K: **Unilateral Biportal Endoscopy Versus Tubular Microendoscopy in Management of Single Level Degenerative Lumbar Canal Stenosis: A Prospective Study**. *Clin Spine Surg* 2021, **34**(6):E323-E328.

14. Deng Y, Yang M, Xia C, Chen Y, Xie Z: **Unilateral biportal endoscopic decompression for symptomatic thoracic ossification of the ligamentum flavum: a case control study**. *Int Orthop* 2022, **46**(9):2071-2080.

15. Heo DH, Lee DC, Park CK: **Comparative analysis of three types of minimally invasive decompressive surgery for lumbar central stenosis: biportal endoscopy, uniportal endoscopy, and microsurgery**. *Neurosurg Focus* 2019, **46**(5):E9.

16. Vernon H, Mior S: **The Neck Disability Index: a study of reliability and validity**. *J Manipulative Physiol Ther* 1991, **14**(7):409-415.

17. Chiles BW, Leonard MA, Choudhri HF, Cooper PR: **Cervical spondylotic myelopathy: patterns of neurological deficit and recovery after anterior cervical decompression**. *Neurosurgery* 1999, **44**(4).

18. Li M, Meng H, Du J, Tao H, Luo Z, Wang Z: **Management of thoracic myelopathy caused by ossification of the posterior longitudinal ligament combined with ossification of the ligamentum flavum-a retrospective study**. *The Spine Journal : Official Journal of the North American Spine Society* 2012, **12**(12):1093-1102.

19. Dworkin RH, Turk DC, Farrar JT, Haythornthwaite JA, Jensen MP, Katz NP, Kerns RD, Stucki G, Allen RR, Bellamy N *et al*: **Core outcome measures for chronic pain clinical trials: IMMPACT recommendations**. *Pain* 2005, **113**(1-2).
